# Descriptive Epidemiology of Amyotrophic Lateral Sclerosis in Japan, 1995-2001

**DOI:** 10.2188/jea.15.20

**Published:** 2005-04-22

**Authors:** Kazushi Okamoto, Gen Kobashi, Masakazu Washio, Satoshi Sasaki, Tetsuji Yokoyama, Yoshihiro Miyake, Naomasa Sakamoto, Heizo Tanaka, Yutaka Inaba

**Affiliations:** 1 Department of Public Health, Aichi Prefectural College of Nursing and Health.; 2 Department of Health for Senior Citizens, Hokkaido University Graduate School of Medicine.; 3 Department of Public Health, Sapporo Medical University School of Medicine.; 4 Project of Scientific Evaluation of Dietary Reference Intakes, National Institute of Health and Nutrition.; 5 Department of Technology Assessment and Biostatistics, National Institute of Public Health.; 6 Department of Public Health, Fukuoka University School of Medicine.; 7 Department of Hygiene, Hyogo College of Medicine.; 8 National Institute of Health and Nutrition.; 9 Department of Epidemiology and Environmental Health, Juntendo University School of Medicine.

**Keywords:** Amyotrophic Lateral Sclerosis, Epidemiology, Mortality, Japan, trends

## Abstract

BACKGROUND: This study was conducted to describe the epidemiologic features of amyotrophic lateral sclerosis (ALS) in Japan by examining annual trends in mortality (1995-2001), and to discuss the background factors possibly responsible for the recent variations in the mortality rate.

METHODS: Trends in both the age-adjusted and age-specific mortality rates of ALS were examined by using the data obtained from the vital statistics of Japan between 1995 and 2001.

RESULTS: There were small increases in the number of ALS deaths (from 1249 to 1400 per year) and the crude mortality rates (from 1.00 to 1.10 per 100,000 population) between 1995 and 2001. The age-adjusted mortality rate of ALS (adjusted using the 1985 model population of Japan) has decreased (from 0.84 per 100,000 population in 1995 to 0.74 in 2001). Age-specific mortality rates have been increasing particularly in the population older than 70 years of age, with the peak in mortality in the 70- to 80-year old age group.

CONCLUSIONS: ALS mortality rates increased proportionally more for elderly population during the study period. Further epidemiologic studies will be needed to clarify the possible background factors contributing to the increase in ALS mortality in the elderly.

Amyotrophic lateral sclerosis (ALS) is a rapidly progressive degenerative disorder that is usually lethal due to a loss of motor neurons in the brain, brainstem and spinal cord.^1^^-^^3^ Patients typically die within 3 to 5 years of onset due to respiratory failure.^1^,^4^ Steadily increasing mortality and incidence rates have been observed worldwide over the past several decades.^5^^-^^20^ These epidemiologic surveys suggested that ageing of the population,^10^^,^^13^^,^^14^^,^^16^^-^^20^ environmental factors,^14^^,^^15^^,^^19^^,^^20^ and geographic distribution^9^^,^^18^^,^^19^ were all related to ALS. In Japan, the yearly number of certified deaths from ALS was first published in 1995 in the annual vital statistics. Since then, no studies have reported the epidemiologic characteristics of ALS in this country. Thus, it is of epidemiologic interest to determine whether or not the ALS mortality rates in Japan are increasing to the same degree as those reported in other countries, because a chronological observation of mortality rates might provide new clues to the etiology of this poorly understood disorder. Thus, we described the epidemiologic features of ALS in Japan by examining the secular trends in mortality (1995-2001).

## METHODS

The numbers of certified deaths from ALS by 5-year age groups were published annually by the Ministry of Health and Welfare of Japan in the period from 1995 to 2001,^21^ according to the International Classification of Disease (ICD-10th) (code:G12). Trends in the crude and age-specific mortality rates were described by standard methods. Age-adjusted mortality rates were calculated by the direct method, using the 1985 model population in Japan as the standard.^22^ The mean age at death was calculated by summing the product of each middle point of the 5-year age groups and the number of deaths in each 5-year group, and then by dividing the sum by the total number of deaths in each year. The age- and sex-specific mortality rates of ALS were calculated as the total number of deaths from 1995 through 2001 divided by the age- and sex-specific number of population in Japan in 2000.

## RESULTS

Table 1 presents the number of ALS deaths per year, sex ratio, crude and the age-adjusted mortality rates, as well as the mean age at death from 1995 through 2001. The number of ALS deaths per year ranged from 1249 in 1995 to 1400 in 2001, and the female to male ratios rose from 1.30 in 1995 to 1.35 in 2001. The average crude mortality rate per 100,000 population from 1995 through 2001 was 1.06, while the crude mortality rate was 1.00 in 1995 and 1.10 in 2001. The age-adjusted mortality rate declined from 0.84 per 100,000 population in 1995 to 0.74 in 2001. The mean age at death increased from 65.7 years old in 1995 to 67.8 years old in 2001, an increase of 2.1 years.

**Table 1.** Mortality of amyotrophic lateral sclerosis in Japan

Year	No. of death	Male/female ratio	Crude mortality*	Age adjusted mortality**	Mean age at death in years
1995	1249	1.30	1.00	0.79	65.7
1996	1342	1.33	1.07	0.84	66.8
1997	1336	1.33	1.06	0.79	66.8
1998	1327	1.33	1.05	0.75	67.0
1999	1382	1.30	1.09	0.76	67.3
2000	1371	1.34	1.08	0.73	68.9
2001	1400	1.35	1.10	0.74	67.8

*: Rate per 100,000 population

**: Rate per 100,000 population, adjusted by the 1985 population in Japan.

**Figure 1.**
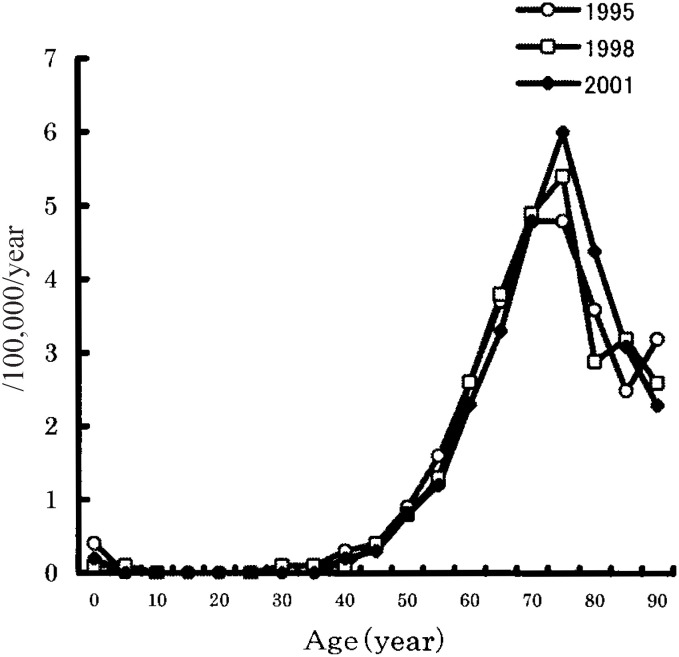
Age-specific mortality rates of amyotrophic lateral sclerosis (ALS) in Japan, in 1995, 1998, and 2001.

**Figure 2.**
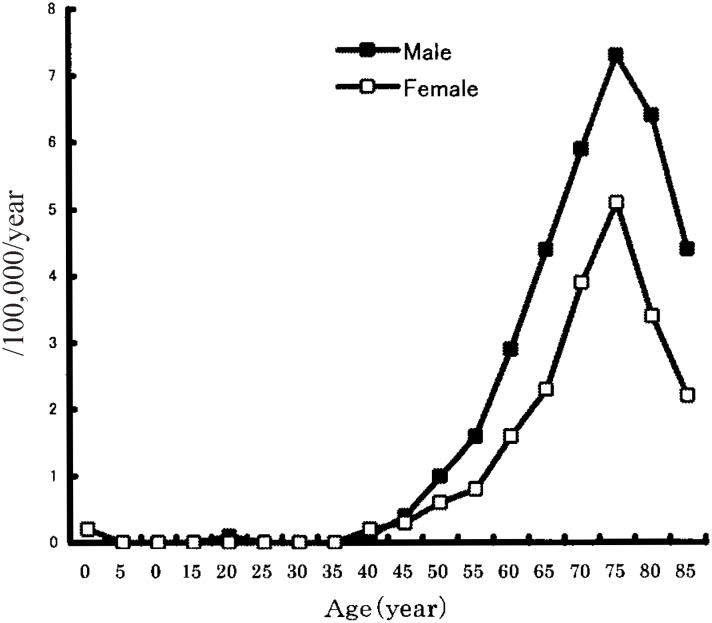
Age-specific mortality rates of amyitrophic lateral sclerosis (ALS) by sex in Japan, 1995-2001.

Figure 1 shows the age-specific mortality rates of ALS in 1995, 1998, and 2001. The mortality rate peaked in the range of 75 to 79 years of age. Between 1995 and 2001, ALS mortality decreased among those younger than 70 years, whereas it increased in those aged 70 or older. Figure 2 shows the age-specific mortality rates of ALS from 1995 through 2001 by sex. The mortality was higher for men than for women in the population older than 45 years of age.

## DISCUSSION

In the present study, we summarized the trends in the number of ALS deaths and ALS mortality rates from 1995 to 2001 in Japan. The present study provides the following major findings: (1) the number of ALS deaths and the mean age at ALS death steadily increased from 1995 through 2001; (2) age-specific ALS mortality rates decreased among those younger than 70 years, whereas it increased in those aged 70 or older; and (3) the ALS age-adjusted mortality rate decreased from 1995 through 2001. To our knowledge, this is the first study to indicate a decline in the ALS age-adjusted mortality rate in Japan. The main epidemiologic feature revealed in this study is that mortality rates increased proportionally more for elderly persons during the study period. With an increase in ALS mortality having been observed among the elderly, the following factors could be taken into consideration: (1) aging of the population, (2) the increase in ALS incidence in the elderly, and (3) the increase in survival time. In terms of item (1), increasing ALS mortality has recently been proposed to pose a growing risk due to the ageing of the studied population.^11^^,^^12^^,^^15^ Statistics on receiving financial aid for the treatment of intractable disease^25^ indicate that the peak number of incident patients shifted towards an older age group in the period 1984-1997. In the present study, moreover, the rate of increase in ALS deaths among those aged 65 and older (136%) was higher than in the general population older than 65 years (125%) from 1995 through 2001. However, the rate of increase in ALS deaths was above that in the general population for the same age group. Thus, the increase in ALS mortality cannot always be related solely to an increase in age in the general population. In terms of item (2), statistics on receiving financial aid for the treatment of intractable disease^25^ indicate that the peak in the proportion of age-specific new ALS patients to the total number of ALS patients has steadily shifted towards increasing age between 1984 and 1997, implying an increase in the ALS incidence among the elderly. In Japan, the diagnostic criteria for ALS have undergone no major changes from 1992 (the first version) to 2000 (the revised version which added electrophysiological diagnosis). However, no remarkable change in the number of ALS death or the mean age of patients at diagnosis was observed in 2000. This finding suggests that the increase in the elderly population may reflect a genuine increase in incidence rather than an improvement in diagnostic accuracy and case ascertainment. In terms of item (3), the introduction of bi-level positive airway pressure (Bipap)^26^^-^^28^ and the use of Riluzole,^®^^29^ which inhibits the advance of ALS, are reported to prolong survival time significantly. Accordingly, it is quite likely that an increased number of ALS deaths in the elderly could result from the prolongation of survival time. However, the magnitude of the effect of prolongation of survival time is unclear in the present study.

In conclusion, we found that age-adjusted mortality rates of ALS had decreased during the study period, and the mean age at death had increased. The increase in ALS mortality in the elderly seems to be explained by an increase in ALS incidence for the index age group. Further epidemiologic investigations are required to clarify the possible background factors contributing to the increase in ALS incidence in the elderly.
